# Tax promotes the expression of the alternative spliced isoform CD44v10 by slowing down the elongating RNA polymerase II

**DOI:** 10.1186/1742-4690-11-S1-P115

**Published:** 2014-01-07

**Authors:** Morgan Thenoz, Céline Vernin, Christiane Pinatel, Nicolas Nazaret, Joel Lachuer, Antoine Gessain, Didier Auboeuf, Eric Wattel, Franck Mortreux

**Affiliations:** 1 Oncovirologie et Biotherapies, UMR5239 CNRS/ENS Lyon/UCBL/HCL, Hopital Pierre Benite, Lyon, France; 2 Oncovirologie et Biothérapies, Centre Léon Bérard, Lyon, France; 3 ProfileXpert, Neurobiotec Service, Bron, France; 4 Unit of Epidemiology and Physiopathology of Oncogenic Viruses, Department of Virology, Institut Pasteur, Paris, France; 5 Institut National de Santé et de Recherche Médicale U590, Centre Léon Bérard, Lyon, France

## 

Deregulation of gene expression is the hallmark of HTLV-1 infection, involving a complex interplay between Tax and host-cell transcription machinery. It has been recently shown that changes in promoter structure and occupation impinge on both transcription and alternative splicing (AS) by modulating the RNApolII elongation rate. These data prompted us to examine whether Tax could induce changes in alternative splicing of cellular genes. The exon expression profiling of 293T-cells expressing or not Tax were investigated using Exon Chip Human microarray. The exon content of 857 genes differed between the two cell-categories, among which only 309 genes were found differentially expressed at the whole transcriptional level. AS events were validated by exon-specific RT-PCR experiments. Of these, we evidenced that Tax-induced NF-kB-dependent transcription of CD44 resulted in the inclusion of variable exon v10 (CD44v10) in mature transcripts. CD44 variants are described in a wide variety of cellular functions including lymphocyte activation, migration, and tumor metastasis. CD44v10 mRNA expression was significantly higher in HTLV-1-infected than uninfected clones deriving from HTLV-1 carriers. The protein expression of CD44v10 at the plasma membrane of Tax+293T-cells was detected by FACS analysis. At the mechanistic level, qChIP analysis of CD44 gene occupancy showed that Tax-induced splicing of CD44v10 coincides with a significant enrichment of both RNApolII and Tax at the region encoding variable exons, indicating that Tax interferes with the elongation rate of RNApolII. Together, these results unmask new mechanisms that connect Tax, transcription and alternative splicing with the HTLV-1-induced transcriptome reprogramming of CD4+ T cells.

